# Opportunities to Harness High-Throughput and Novel Sensing Phenotypes to Improve Feed Efficiency in Dairy Cattle

**DOI:** 10.3390/ani12010015

**Published:** 2021-12-22

**Authors:** Cori J. Siberski-Cooper, James E. Koltes

**Affiliations:** Department of Animal Science, Iowa State University, Ames, IA 50011, USA; jekoltes@iastate.edu

**Keywords:** feed efficiency, precision technologies, novel phenotypes

## Abstract

**Simple Summary:**

Sensors, routinely collected on-farm tests, and other repeatable, high-throughput measurements can provide novel phenotype information on a frequent basis. Information from these sensors and high-throughput measurements could be harnessed to monitor or predict individual dairy cow feed intake. Predictive algorithms would allow for genetic selection of animals that consume less feed while producing the same amount of milk. Improved monitoring of feed intake could reduce the cost of milk production, improve animal health, and reduce the environmental impact of the dairy industry. Moreover, data from these information sources could aid in animal management (e.g., precision feeding and health detection). In order to implement tools, the relationship of measurements with feed intake needs to be established and prediction equations developed. Lastly, consideration should be given to the frequency of data collection, the need for standardization of data and other potential limitations of tools in the prediction of feed intake. This review summarizes measurements of feed efficiency, factors that may impact the efficiency and feed consumption of an animal, tools that have been researched and new traits that could be utilized for the prediction of feed intake and efficiency, and prediction equations for feed intake and efficiency presented in the literature to date.

**Abstract:**

Feed for dairy cattle has a major impact on profitability and the environmental impact of farms. Sustainable dairy production relies on continued improvement in feed efficiency as a way to reduce costs and nutrient loss from feed. Advances in breeding, feeding and management have led to the dilution of maintenance energy and thus more efficient dairy cattle. Still, many additional opportunities are available to improve individual animal feed efficiency. Sensing technologies such as wearable sensors, image-based and high-throughput phenotyping technologies (e.g., milk testing) are becoming more available on commercial farm. The application of these technologies as indicator traits for feed intake and efficiency related traits would be advantageous to provide additional information to predict and manage feed efficiency. This review focuses on precision livestock technologies and high-throughput phenotyping in use today as well as those that could be developed in the future as possible indicators of feed intake. Several technologies such as milk spectral data, activity, rumen measures, and image-based phenotypes have been associated with feed intake. Future applications will depend on the ability to repeatably measure and calibrate these data across locations, so that they can be integrated for use in predicting and managing feed intake and efficiency on farm.

## 1. Introduction

Sustainable agricultural practices are increasingly important to feed a growing world as animal-sourced protein is required for proper cognitive development in children [1]. A major contributor to sustainability in the dairy cattle industry is feed intake and efficiency, as it impacts economic and the environmental impacts. Feed has the largest economic impact on dairy farm profitability, at more than 40% of the expenses in the production of milk [2].

Improvements in feed efficiency will also positively impact the environment by reducing greenhouse gas emissions of cattle and manure (Food and Agriculture Organization of the United Nations) [3] as well as land requirements for manure disposal and water needs [4,5]. Improvements in production efficiency and management practices have already reduced the carbon, water, and land use footprints of the dairy industry [5,6,7]; however, additional improvements are needed to reach future sustainability goals (e.g., Net Zero Initiative [8]). A next step could involve the use of precision sensing data to monitor sustainability metrics as part of SMART farms [9] that use data to inform producers on how to be more efficient on dairies.

New methods and technologies are needed to monitor feed intake in the dairy industry since current technologies that measure individual feed intake are not practical on commercial farms. The methods used today to predict feed efficiency utilize costly feed intake systems on research farms to track individual feed intake, milk and component data, body weights that are modeled with advanced statistical models [10,11]. Sensing technologies may be useful as indicators of feed intake as they are portable, and some are already available on commercial dairies today. Likewise, milk testing data are another information resource the use of which could be expanded to help predict feed intake. The objective of this review is to discuss new opportunities to use data from state-of-the-art technologies such as sensing technologies and high-throughput lab data to monitor feed intake and other factors known to impact various definitions of efficiency.

## 2. Defining Feed Efficiency

There is no one single definition or set of traits that have been agreed upon to define feed efficiency. This may be one reason why feed efficiency traits have yet to be monitored directly in dairy cattle in most countries. Two other important factors are (1) the high cost and labor to collect individual feed intake measurements; and (2) limited availability of measurements on correlated traits (e.g., body weight). To date, most individual animal feed efficiency data have been collected on research facilities. This section will describe common feed efficiency definitions, component traits and genetic tools developed to manage feed efficiency in dairy cattle herds today. (For more information, see Pryce et al. [12,13].)

### 2.1. Dry Matter Intake

Feedstuffs can broadly be separated into water and nutrient sources, which can be measured as moisture and dry matter. Dry matter is simply everything remaining after water content has been removed. Since moisture content can vary considerably across diets, dry matter is often considered a fairer measure of the nutritional content of a ration [14]. An obvious way to minimize feed expense would be to decrease dry matter intake (DMI), without a reduction in milk production. Genetic correlations between DMI and milk production range from 0.44 to 0.94, indicating selection for decreased DMI is expected to decrease milk production if these correlations are not accounted for in breeding value estimation [15,16,17]. Heritability estimates for DMI range from 0.17 to 0.60 and vary depending on the stage of lactation, study location, and parity, indicating genetics have a significant contribution in the variability of DMI [18,19,20].

### 2.2. Gross Feed Efficiency

Feed conversion ratio, also known as gross feed efficiency (GFE), is the proportion of output to input, or the amount of milk output produced per one unit of feed intake in lactating dairy cattle. Milk output and feed input can be defined in many ways, which may lead to inconsistencies [21]. Nonetheless, use of GFE is desirable in the sense that it is relatively easy to measure, and its concept is easily explained and understood [22]. Reported heritabilities of GFE are moderate, ranging from 0.14 to 0.37 [19,23]. The high genetic correlation between milk yield and GFE (0.88–0.95) indicates GFE is already indirectly being selected upon [12,24].

### 2.3. Residual Feed Intake

Residual feed intake (RFI) is defined as the difference between an animal’s actual feed intake and expected feed intake, accounting for the animal’s energy sinks [25,26]. In RFI, energy sinks include milk production (e.g., energy corrected milk and fat-corrected milk), body weight (BW) and BW fluctuations [11,13,15,27]. An advantage of RFI is that it is defined mathematically to be phenotypically uncorrelated to milk production traits. Heritability estimates for RFI vary by age, days in milk (DIM), and energy sinks, ranging between 0.02 and 0.38 [12,23,27,28,29,30]. Few studies have obtained reliable estimates of genetic correlations with other economically important traits [22].

### 2.4. Feed Saved

Feed saved is defined as the reduced feed intake (i.e., the actual amount of feed saved) between an animal’s actual feed intake and the predicted intake-based accounting for milk production and maintenance requirements using BW and RFI [12]. A breeding value for feed saved has been developed for use in the U.S. dairy industry for genetic selection, where the RFI component has a heritability of 0.14 [31]. Relationships with other traits are currently under investigation.

## 3. The Application of Precision Technologies in the Dairy Industry Today

**Figure 1:**
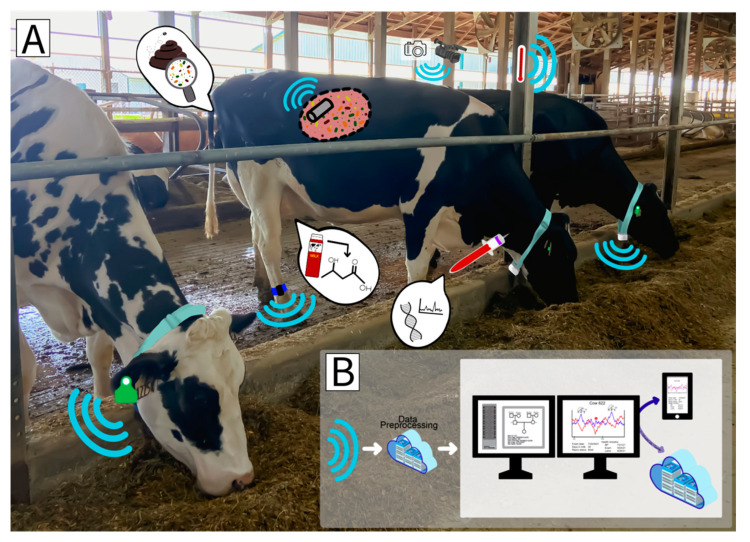
Overview of precision technologies, high-throughput assays and genetic data that could be applied commercially in the improvement of feed intake and efficiency. (**A**). Sensing technologies in use today include wearable sensors, such as milking collar, leg band and ear tag-based sensors with accelerometers, temperature sensors, and proximity sensors; image-based monitoring systems; internal sensors (e.g., rumen boluses); and environmental sensors which can monitor temperature and humidity. High-throughput assays include milk component data derived from mid-infrared spectral data, capturing small molecules in milk. Future technologies may detect such small molecules in the blood. Additional molecular data could potentially be obtained from the rumen or fecal microbiome. (**B**). All of this information could be integrated with genetic and genomic information that is already used today to predict breeding values for genetic improvement of feed efficiency.

Precision livestock farming (PLF) can be defined as using the principles and technology of process engineering for the management of livestock [32]. Simply put, PLF is the use of real-time sensors, devices, and technologies to monitor livestock in an automated fashion. Devices used in such monitoring are often referred to as precision livestock technologies (PLT). As cow numbers are continuing to increase and labor levels remain unchanged, PLT have the potential to provide large benefits in the monitoring of animal performance, behavior, and health [5,33]. PLT, as well as the often forgotten high-throughput assays, will likely provide new or hard to measure information needed to identify the most efficient cows, reduce health problems, and improve farm management. An overview of cow-level data (PLT, high-throughput phenotypes and genetic or molecular information) either in use today or of interest for the future to predict feed intake is shown in Figure 1.

### 3.1. Milk Monitoring Systems

As milk monitoring systems have advanced, the ability to measure a large number of traits at each milking has become possible. Conventional and automated milking systems (AMS) can detect individual milk weights, components, somatic cell scores (SCC), conductivity, flow rates and many more measures. The wealth of data regularly recorded via milking systems is often underutilized. It is likely that milk information will be of great benefit in the determination of feed intake and efficiency [34,35]. For example, mid-infrared spectroscopy (MIR) is used to routinely monitor milk components at standardized testing laboratories. Some milking systems are including in-line MIR or similar spectral sensors that can monitor a variety of different milk components and compounds today in hopes that milk molecules will be informative about a cow’s energy balance, fertility, health and potentially efficiency. The ability of MIR and related technologies to detect molecules in milk on a daily basis could be very powerful analytics (e.g., Dórea et al. [34]). However, challenges for these milking systems include limited integration of different sensing data and difficulty in standardization and pooling of data across locations. Moreover, commercial dairy farms often only collect milk samples every four to six weeks. Thus, depending on when an animal calves in regard to the milk testing cycle, key information (e.g., milk-based information around parturition) could be missed. Additionally, since these data are time specific, the sparsity of data reduces the ability to track daily or weekly changes in the spectra that could have application in prediction of other phenotypes (e.g., health and feed intake).

### 3.2. Collar/Halter-Mounted Monitors

Wearable devices mounted on cow collars or halters have been developed to monitor traits such as activity, rumination and feeding and drinking time using accelerometer and microphone technologies. Potential benefits of such devices include less sensor loss and expanded use of hardware such as collars that are often already on cows for parlor monitoring. Challenges associated with these technologies include the need for periodic adjustment, replacement (i.e., battery life), and difficulty in calibration, which may impact sensor accuracy or require additional labor.

### 3.3. Leg-Mounted Devices

Devices attached at the leg are most often utilized to monitor animal activity using accelerometers or technologies that track animal location proximity. Such technologies are potentially the earliest form of PLT to be used in the dairy industry, originally for estrus detection applications. Over time, leg-mounted devices have been further developed to serve as a tool to help monitor animal health. Challenges associated with leg-mounted devices include limited access (typically only during milking), and difficulty in calibration [36].

### 3.4. Ear Tag Technologies

Ear tag technologies can track temperature, activity, rumination, feeding behavior, panting time and location. Accelerometers, proximity sensors, and skin temperature sensors are most often applied within these devices. Sensor-based ear tags are most commonly used in the dairy industry for estrus detection but are gaining in popularity for health monitoring [36]. With these technologies already being commonplace commercially, their implementation for use in the prediction and determination of feed intake and efficiency should, in theory, be rather straightforward. There are, however, potential downfalls in the use of ear tags. These include a limited battery life [36], loss of tags, variability in tag placement and difficulty in calibration.

### 3.5. Rumen Boluses

Rumen boluses have been developed to monitor an animal’s activity, rumen temperature and in some cases rumen pH. Rumen boluses have been used on a limited basis on commercial farms, largely due to their cost associated with pH sensing compared to other technologies. Devices that monitor rumen-based activity and temperature alone are considerably more affordable and have longer battery life. Battery life of rumen boluses that sense pH are substantially shorter than other sensors, lasting only approximately six months [37]. Further retrieval of these devices is not possible, and calibration of the sensors is challenging.

### 3.6. Image-Based Technologies

Recently, several new image sensing technologies have become commercially available to monitor animal health and feed intake. Relatively little is known about how these are working in the industry today; however, based on previous research there is incredible potential for image data to revolutionize the industry [38,39]. The major advantages include the ability to have contact-free monitoring of animals and the ability to observe new, previously unknown behavioral phenotypes. Image device challenges include keeping camera lenses clean to permit constant data flow, access to power or internet network to record phenotype data and relatively limited information about calibration of these devices. Acquisition of digital phenotypes from images through computer vision is an extensive area of research. Additional information about considerations in the application of computer vision and state-of-the-art machine learning methodologies employed is available in the following reviews [40,41,42].

Sensing technologies are evolving rapidly beyond the more commonly used technologies discussed above. Given the various applications for these sensors, the logical next step would be to start monitoring feed intake and other traits impacting the efficiency of dairy cattle. The following sections of this review will be efficiency trait centric, with specific discussion on sensing technologies as indicators of the various subcomponents or composite measurements that impact feed efficiency. The next section will discuss commonly used technologies, followed by under-utilized and cutting-edge sensing data, which may revolutionize our ability to manage dairy feed efficiency but have yet to be implemented in commercial dairies.

## 4. Traits and Environmental Variables Associated with Feed Intake and Efficiency

Numerous factors impact how much feed an individual animal consumes and its feed efficiency. Factors with potential impacts can be divided into two overarching categories: individual animal variation in traits and environmental factors, including management decisions. Trait examples include energy sinks such as milk production and maintenance energy, as well as behavior. Additional variables that impact feed intake are described below. Sensors that may directly monitor or be correlated with feed intake and efficiency are reported in Table 1.

### 4.1. Energy Sinks

#### 4.1.1. Milk Production Measurements

Milk, fat, and protein yield are important metrics in understanding variation in an individual animal’s efficiency, as feed usage per unit of output is important. Research indicates that milk and protein yield [59] are likely of most importance in evaluating feed efficiency. Modern milking systems (conventional and AMS) routinely measure many traits at milking, such as milk weights, components, and conductivity (an indicator of SCC and mastitis). Milk yield, components (e.g., fat, protein, and lactose), and SCC were previously associated with feed efficiency [29,60]. Prediction equations using MIR data (i.e., data used in the determination of milk components) have been developed for energy balance [35], metabolic status [61], RFI [43,44] and DMI [34,44,45]. The range in reported accuracies is wide; however, it appears the utilization of MIR data to predict feed efficiency could be greatly beneficial. Importantly, if MIR information is to provide accurate estimates, effective calibration of a prediction equation would be required. This would need to include some means of accounting for location and differences in MIR machines, so that data could be compared across testing locations and farms [62], MIR measurement technologies [63,64,65] and feeding systems (i.e., high- vs. low-concentrate diets [35]). Another major hurdle in the commercial application of MIR for the prediction of feed intake is increasing the frequency of measurement, as such discussed previously. For more comprehensive information on MIR and other infrared spectrums to predict complex traits, please see Bresolin and Dórea [66].

#### 4.1.2. Maintenance of Body Weight and Condition

Dairy cattle have a baseline net energy requirement for maintenance. Feed used for maintenance supports essential functions for life, such as blood circulation and respiration [29]. In the instance that a cow eats at maintenance level and does not produce milk, her GFE would be 0. Additional energy consumption above maintenance can be utilized to produce milk or converted to body tissues. As an animal eats an increasing amount, the proportion of total feed intake utilized for maintenance diminishes (i.e., the dilution of maintenance), resulting in increased efficiency. Body weight is important in understanding variability in feed intake and, for this reason, routinely used in genetic evaluations (e.g., metabolic body weight and ∆ body weight). Automated scales connected to milking systems and camera-based imaging systems may allow for monitoring of body weight over time. Further, milk component and MIR data may provide useful information about changes in the catabolism of body fat reserves during negative energy balance.

### 4.2. Novel Sensors and Phenotypic Measures That May Aid in Predicting Feed Intake—Looking toward the Future

#### 4.2.1. Activity

Variation in individual animal activity likely influence feed efficiency since energy for movement and heat production alter maintenance requirements [67]. Conflicting associations between activity and feed efficiency have been reported [52,53,68]. Several recent studies have identified relationships between feed intake and activity [54,55]. Moreover, several studies conducted in pigs have identified differences in activity between low and high RFI animals [69,70,71]. Activity data appear to be a promising phenotype for use in predicting feed intake [54].

#### 4.2.2. Thermoregulation and Heat Stress

Thermoregulation is an important contributor to feed efficiency of cattle [67,72]. This is driven by the fact that animals with higher core body temperatures utilize more energy for heat production than their contemporaries with lower core body temperature [72,73]. In fact, Shuey et al. [74] found that fasting heat production accounted for more than 70% of the variation in maintenance requirements in cattle. Moreover, evaporative heat loss is the primary route of energy loss in ruminants, and this is largely regulated by respiratory rate [67]. Thus, respiration rate could be correlated to feed intake. Body surface temperature may be another measure relevant to feed intake, which can be measured by infrared thermography. More efficient dairy cattle were found to have numerically lower surface temperatures of the lower rear leg than less efficient cows. In addition, regression analysis of leg and paralumbar fossa surface temperature tended to explain variation in RFI (*p <* 0.10 [15]). Similarly, lower skin temperatures of beef cattle have been associated with lower RFI values (i.e., more efficient [75]). Further, heat stress is known to effect feed intake and milk production [76,77,78,79], and therefore likely impacts measures of feed efficiency. Research also indicates that heat stress effects post-absorptive carbohydrate metabolism [76], which has been suggested to play a key role in the efficiency of an animal [67,80,81]. Therefore, sensing-based measurements of heat stress are likely important for understanding variability in feed intake and efficiency.

#### 4.2.3. Rumen Characteristics

Rumen characteristics are important contributors to feed efficiency [82]. A host of measurements from the rumen could be evaluated with sensors or high-throughput assays, including rumination, pH, temperature, and microbial content. It is well established that rumen pH is affected by the diet [83,84]; however, individual cow variation is still observed likely due to variable buffering and acid absorption [84,85]. Acidotic conditions cause health events and reduce milk production [84,85,86], thus reducing feed efficiency. Water and feed intake impact rumen temperature [37]. Fischer et al. [57] found that more efficient lactating Holsteins showed decreased variability in rumen temperature when correcting for drinking events, indicating it will be important to consider eating and drinking bouts in further research of this measure. Rumination time and feed intake are directly related (i.e., more feed consumption should result in increased rumination time), and therefore research into rumination as an indicator of feed intake is warranted.

#### 4.2.4. Microbiome

The microbiome in the gastrointestinal tracts of cattle is key in the ability of ruminants to digest and absorb nutrients from plant mass [87,88,89,90,91,92]. Host genetics and factors such as diet composition, rumen pH, age and sex of an animal influence microbiome composition [91,93,94,95]. Multiple studies have identified relationships between rumen microbiome composition and the efficiency of energy conversion [87,88,89,91,92,93,95,96]. Previous research determined that more efficient Holstein Friesian cows have microbiomes that are lower in richness and diversity (i.e., specie number and bacteria within a species, respectively) and higher in dominance of microbes. With this, the microbiomes exhibited less complexity and an increased specialization to support the energy requirements of the host [89]. Importantly, studies have shown there are heritable elements in the rumen microbiome associated with host traits such as DMI and RFI [92,93,95]. In addition to differences in the rumen microbiome associated with feed efficiency, a study involving the fecal microbiome of cattle found bacterial operational taxonomic units that were unique to RFI groups [97]. Elolimy et al. [98] found differences in hindgut microbiome of more- and less-efficient Holstein calves at birth and during the pre-weaning period. Such findings indicate that microbial communities beyond those in the rumen influence how efficiently an animal utilizes feed for production. There are not yet technologies to measure the rumen or other microbiomes, though such sensors could be valuable to monitor health and efficiency of animals.

### 4.3. Management and Nutrition Factors Affecting Feed Intake and Efficiency

The ability to predict and mange feed efficiency through precision feeding is an attractive approach to reduce costs and environmental impacts of dairy cows. Nutrition is a well-known factor impacting feed intake and efficiency (i.e., diet composition, feedstuff processing procedures, and nutritional grouping of animals). VandeHaar and St-Pierre [99] have described how increased fiber digestibility results in increased milk production and thus increased GFE. Feed particle size, processing and changes in the diet also impact rumen health and subsequently efficiency. Numerous other measurements and attributes of feed could be monitored or examined on a regular basis by sensors and sensing technology to aid in the determination of how efficiently an animal utilizes feed to produce product. Feed level information could be detected by sensors and potentially integrated into precision feeding models. Precision feeding would prescribe feed intake levels on an individual cow basis, based on DIM, milk and component production, feedstuff prices and the predicted variation in traits associated with feed efficiency measures [100]. Precision feeding has been shown to increase both milk and fat-corrected milk yield, as well as physical feed efficiency [101]. Models have been developed to predict dry mater in take in lactating dairy cows [10,102], but additional information will likely be added in the future to improve predictions, thanks to new studies and information collected through precision sensing technologies.

## 5. Monitoring the Impacts of Stress and Illness on Feed Efficiency

When a cow is physiologically stressed, its maintenance requirements increase to mitigate the source of stress. In order to meet this higher maintenance requirement, nutrient partitioning may shift, ultimately impacting feed efficiency [60,103,104]. An example of such partitioning may include using energy normally devoted to production to instead support the immune system [105]. The redirecting of energy away from production could result in decreased milk production and a reduction in the dilution of maintenance, ultimately leading to decreased feed efficiency [60,106]. As of today, there has been little research on the direct impact of specific diseases/illnesses on feed intake and efficiency in dairy cattle. Briefly, research has shown that when undergoing a bout of mastitis, energy output and input are decreased, but the decrease in output outweighs the decrease in input, resulting in decreased GFE [107]. Similarly, a decrease in intake and production has been observed when an animal is lame [108,109].

### Identification of Stress and Disease

A major application of PLF and automated sensors commercially is attributed with providing information for the detection of health events and aiding in management decisions [110]. The majority of research thus far has focused on the identification of mastitis and lameness with sensors, though there have been efforts to identify metabolic diseases. For more information on PLT to detect health events, please see King et al. [111], Maltz [112]. Numerous studies indicate illness is associated with changes in milk production which can be monitored by milk monitoring systems, collar systems, leg-mounted devices, ear tags and rumen boluses. A future need is to quantify losses in feed efficiency due to illness and identify animals who are more resilient to production losses during illness, as these animals will be more feed efficient. Sensing technologies may be able to help identify robust individuals who are more efficient, due to their ability to overcome illness and bounce back more quickly to previous milk and component productivity levels.

## 6. A Closer Look at Feed Intake Prediction Methods Integrating Sensing Technologies

The following passage summarizes five studies that have predicted feed intake with sensing or high-throughput phenotyping technologies. These studies apply, and in some cases compare multiple methodologies of predictive ability. The quality of prediction was evaluated using measurements of precision and accuracy (e.g., concordance correlation coefficient; CCC, coefficient of determination; R^2^ and cross-validation or leave-one-out (LOO) validation methods), the amount of variance in DMI explained (e.g., R^2^), and the amount of residual error unexplained (root mean square error of prediction; RMSE).

### 6.1. Martin et al.: Prediction of DMI and RFI Using Activity and Blood Metabolite Data

The objective of Martin et al. [54] was to predict feed intake utilizing four datasets, each building upon the information used in the previous model. The initial dataset included milk traits (i.e., yield and components), the next added body size-related traits, the following also included behavior traits recorded via sensors, and lastly blood metabolite measurements were incorporated. Data were recorded on 124 mid-lactation (50–200 DIM) Holstein cows in two replicates (62 cows per replicate). In addition to exploring how the addition of novel sensing phenotypes affected prediction, four types of prediction models were assessed. These included multiple linear regression (MLR), partial least squares regression (PLSR), artificial neural networks (ANN), and stacked ensembles (SEB). LOO cross validation was applied for all methods; however, 5-fold cross validation algorithms were used prior to LOO cross validation in models utilizing hyperparameters, in order to tune the models. Regarding DMI, the largest improvement in predictive performance was observed by adding body size traits to milk recording variables. The highest prediction accuracy for DMI was observed for the MLR model using the dataset including sensor-based behavior traits (R^2^ = 0.82; CCC = 0.90; RMSE = 1.68 kg/d). Addition of blood metabolites to the model generally decreased accuracy and precision (e.g., ANN: R^2^ = 0.79 vs. 0.81; CCC = 0.88 vs. 0.90; RMSE = 1.78 vs. 1.64), except when MLR was used, and no difference was seen compared to the sensor trait model. When predicting RFI, performance was poor regardless of dataset or predictive method used (best RFI R^2^ = 0.13 and CCC = 0.29 for MLR with the full dataset including metabolites).

### 6.2. Dórea et al.: Addition of MIR Data into DMI Predictions

The objective of Dórea et al. [34] was to predict feed intake utilizing MIR and behavior data in addition to energy sinks (i.e., milk yield, metabolic body weight, milk components) with ANN versus PLSR models. Feature selection was applied to identify wavelengths from MIR data by Bayesian network (BN) and only selected wavelengths were modeled. Nearly 1280 milk samples collected from 308 animals were used and models were validated using an external dataset. Generally, ANN performed better than PLSR models. The addition of the BN selected spectra to milk components (i.e., fat, protein, lactose) in the ANN minimally improved predictive performance (R^2^ = 0.54 vs. 0.53; CCC = 0.73 vs. 0.72; RMSE = 2.71 vs. 2.81 kg/d). The best predictions resulted from the inclusion of duration of time at the feed bunk (i.e., feeding time) and the BN selected MIR in the ANN model (R^2^ = 0.70; CCC = 0.83; RMSE = 2.15 kg/d).

### 6.3. Shetty et al.: Application of MIR Data to Predict DMI and RFI

The objective of Shetty et al. [44] was to predict DMI and RFI with MIR data. MIR data from 140 cows (97 Holsteins and 43 Jerseys; 1044 weekly average DMI records) were utilized in PLSR prediction models for weekly average DMI and RFI. Validity of the prediction models were assessed by either (1) randomly leaving out 20% of records; (2) randomly leaving out 20% of cows; or (3) randomly leaving out one cow (i.e., LOO) for validation and using the remaining data for training. Highest accuracies were obtained when utilizing method (e.g., PLSR using milk yield, weight, and MIR: R^2^ = 0.81 vs. 0.77 and 0.42; RMSE = 1.49 vs. 1.65 and 2.07 kg). Moreover, models were developed within and across lactational stages (based on DIM). The prediction model for DMI including only MIR resulted in an R^2^ of 0.30 and RMSE of 2.91 kg. Combining MIR with milk yield and weight resulted in an R^2^ of 0.81 and RMSE of 1.49 kg. The prediction of RFI changed throughout lactation, with early lactation having the highest accuracy compared to across-, mid- or late-lactation (Early: R^2^ = 0.46; RMSE = 1.70; Mid: R^2^ = 0.08; RMSE = 1.35; Late: R^2^ = 0.14; RMSE = 1.33; Across: R^2^ = 0.24; RMSE = 1.46). The most important spectra contributing to DMI and RFI prediction were those related to fat, protein, and lactose peaks. Similar results were obtained by including MIR predicted fat, protein, and lactose instead of MIR data.

### 6.4. Lahart et al.: Milk MIR and Fecal Near-Infrared Spectral (NIR) Data for DMI Prediction

The objective of Lahart et al. [45] was to evaluate the use of MIR and fecal NIR data to predict DMI. Linear regression and PLSR using 7 datasets were evaluated for the prediction of DMI. Datasets included information related to lactation (production, stage, and parity), MIR from milk and NIR from fecal samples. Data were recorded from over 450 animals (337 Holstein-Friesians and 120 Jersey cross Holstein Friesians; 1074 records). Initially, split-sample (i.e., removing every 20th sample) cross validation was conducted on all PLSR models to determine the minimum number of partial least squares factors needed to obtain the lowest RMSE. Within herd validation and across herd validation was utilized for all prediction equations. Within herd validation resulted in higher accuracies (e.g., model including MIR: R^2^ = 0.76 vs. 0.64; RMSE = 1.51 vs. 1.59 kg). The addition of MIR improved prediction slightly (R^2^ = 0.64 vs. 0.60; RMSE = 1.59 kg vs. 1.68 kg). Highest accuracy was achieved by including both the MIR and NIR data (R^2^ = 0.68; RMSE = 1.52 kg).

### 6.5. De Souza et al.: Prediction of DMI with Energy Sink and Body Size Data

The objective of de Souza et al. [10] was to develop prediction equations for DMI to compare against the recommended Nutrient Requirements in Dairy Cattle (NRC) guidelines. A mixed effects model and a non-linear mixed model were used to predict DMI. Over 31,600 weekly observations were obtained from about 2800 cows. The prediction model included DMI, milk energy, change in body weight, body condition score, height, DIM, parity and two-way interactions with parity, and other systematic effects (i.e., location, study, diet and cow). The non-linear model consisted of a two-step approach. The first step was a linear model component to predict DMI in mid-lactation cows (DIM 76–175) and the second step was a non-linear adjustment for DIM using data across the whole lactation (DIM 0–368). The non-linear model was also compared to a linear model with a 4th-order polynomial for DIM using data from the entire lactation (DIM 0–368). A 5-fold across-study cross validation was used. Accuracy and precision measures for the best-fitting model are as follows: CCC = 0.72, RMSE = 2.89. Researchers compared predicted DMI using their model to the 2001 NRC and found their model had smaller mean bias and RMSE and higher CCC (i.e., improved predictive ability).

These studies demonstrate the potential to predict feed intake with reasonable accuracy (CCC > 0.70) with a variety of data types and methods. Accounting for stage of lactation (DIM) and inclusion of novel data such as activity, metabolites, NIR or MIR improved model predictive performance. Interestingly, prediction of DMI had higher accuracy than the prediction in RFI within Martin et al. [54]. This study also explained the highest percentage of variability of DMI among these studies (R^2^ = 0.82). Feature selection approaches as described in Dórea et al. [34] helped in selecting the most informative spectra, resulting in the highest R2 (0.81) among MIR studies predicting DMI. Several of these studies used machine learning-based methods, but mixed models and regression-based approaches were also successful and, in some cases, outperformed machine learning methods [54].

## 7. The Future: Possible New Phenotypes and Tools for Predicting Feed Intake and Efficiency

As technology and science advance, the number of possible phenotypes and tools for use in the prediction of feed intake and efficiency has become limitless. This begs the question of how do researchers target the most useful technologies, in order to identify solutions efficiently? The answer to this question likely varies between research groups; however, factors such as the ability to apply tools commercially, potential uptake by the industry, and data availability and usability need to be considered. Regarding prediction of feed intake and efficiency, more research is needed to develop algorithms and tools to improve accuracies in commercial farm settings. Predicted feed intake could be used for precision feeding, management, genetics, and culling decisions to improve the sustainability of individual dairy herds, as well as the larger industry. At present, MIR and activity data appear to be the most promising data for inclusion in new prediction models. However, both methods will require more research to determine how to best calibrate and integrate data across locations where they are measured. Development of new technologies will likely need to overcome these same challenges.

### 7.1. Image-Based Phenotyping

A frontrunner technology to provide new feed intake and efficiency related data in the future is image data. Multiple types of camera-based methods have been investigated for use in estimating individual feed intake, body weight and condition score, and detection of gate abnormalities. Types of methods include Light Detection and Ranging (LIDAR) sensing [46], 3-dimensional cameras [39,47,48,49,50,113] and photogrammetry [51]. In the prediction of intake or body weight, these methods utilize various methods to estimate volume or mass and can thus utilize additional algorithms to determine intake from the difference post- and pre-eating bout or the weight of an animal [39,46,47,51]. Body condition score can be predicted through the assessment of contours in key areas of fat deposition, such as around the tailhead and between the hooks and pins [48,49,50]. Lastly, lameness can be detected by assessing back angles and posture, based on the fact that curvature of the back is an indicator of lameness [113,114]. Research thus far indicates that implementation of cameras for on-farm estimates and prediction is feasible; however, some systems may be more advantageous than others for commercial use. For example, some cameras are more sensitive to sunlight and thus careful consideration needs to be given to the placement of them, whereas other systems lack sensitivity to lighting conditions and may be easier to implement [51].

### 7.2. Other Novel Technologies

Additional underutilized technologies that could be developed for use to predict feed intake (including possible measurements), include environmental sensors (heat and cold stress), implantable sensors (proximity to feed, temperature, and activity), sound monitoring (feeding behavior), metabolite profiling (metabolic or immune status), and microbiome profiling (health and feed intake). Technological advances are needed to employ some of these technologies, while others could be used implemented today.

## 8. Overcoming Challenges and Developing Tools to Manage Feed Efficiency

Precision management of feed and feed efficiency on farm has incredible potential to improve dairy sustainability and profitability. The use of precision technologies will help in the development of both day-to-day predictive analytics, as well as genetic management tools. Application of precision technologies will also help spur new research because many of the new automated data collection systems allow measurement of traits that were previously difficult or impossible to measure. However, to truly make use of precision technologies to better manage feed on farm, there are a number of challenges that will require resolution.

A major challenge to implementing feed intake prediction on farm is where the data will come from, and how it will be standardized and calibrated across sampling locations. Clearly, large scale measurement of individual cow feed intake outside of research farms is not feasible. One solution may be the use of sensors and high-throughput assays as proxies. These proxies need to be scalable and robust enough to survive daily activities on a commercial dairy farm. Sensing devices are also needed to monitor fluctuations in body weight, which is important to understanding energy utilization in cows. Automated milking parlor returns with scales, AMS and cameras are all potential solutions to monitoring changes in bodyweight. To utilize proxy data, there will need to be systems to collect and clean data. Existing data infrastructure within the dairy industry may be able to provide this resource. Data quality and calibration standards are also a continuing challenge with sensor data. Entities such as International Committee for Animal Recording (ICAR) could help to determine the accuracy, precision, and reproducibility of data from automated sensing systems. Proxy data, such as milk testing, needs to be observed at a regular enough frequency to be informative and also cost effective. One challenge with milk testing is that many producers are no longer milk testing due to cost. Future inline sensors within milking systems or updates that reduce the cost of milk testing may help overcome this problem. Another critical challenge will be defining how to use technologies across different points during lactation (i.e., DIM) given the variability in metabolic demands on the cow overtime.

Often, the needs of those developing technologies and the ability to utilize sensor data are at odds. For example, proprietary software can make it challenging to obtain, develop new uses for and integrate data from proprietary sensors. More research is needed in this area and incentives are needed for industry to collaborate in the development of innovative new tools that benefit both industry and producers. Automated technologies also need to be economical, both for companies to produce and producers to afford. Any analytics developed from precision technologies need to be understandable. For example, RFI is a helpful trait for understanding feed efficiency, but extremely difficult to explain to producers compared to other traits like feed saved. Ideally, such jargon would be tested with producers. Data overload is another challenge, in which producers can be overwhelmed with information or not know what the actionable outcome is for sensors. A final consideration of note is that researchers can develop all kinds of wonderful analytics and technology, but if it’s not usable and industry is not willing to adopt it, then sensors and sensing technologies will be of no impact in the dairy industry. Moreover, if society and customers of the dairy industry view these new technologies as invasive or impersonal over being aids and precision health care tools to enhance animal care and welfare, then these precision technologies will not be able to help improve feed efficiency or other metrics related to dairy sustainability.

## 9. Conclusions

Sensors and other high-throughput assays are rapidly changing the dairy industry by providing new information and innovative approaches to monitor animal fertility and health. We believe it is likely that sensors will eventually allow monitoring of individual feed intake on commercial farms, enabling management decisions and selection for more feed efficient dairy cattle. The key next step will be in the development of analytics that are truly actionable and simple for dairy producers to utilize. It is well established in research that body weight, milk energy and feed nutrients impact efficiency, but there are several new technologies such as MIR and animal behavioral data (e.g., activity) that could enhance our predictions. It is likely new information measured by sensors may help track differences in energy sinks, behavior, genetic or environmental factors that aid in our understanding of feed and nutrient efficiency in the future. Use of these new precision dairy tools will depend on the cost, ease of application, durability and replication on farm. Sensing technologies are still in the hype cycle today, where much of what can be achieved is yet to be realized despite some early successes. The next steps will require more calibration and standardization of sensing data, continued identification of new traits associated with feed intake, and development of prediction equations and validation in independent populations. Once new, valuable pieces of information are identified, then focus can shift to how to measure them on farm and integrate them into existing models. Integration of multiple sources of data will likely lead to the most informative analytics for management.

## Figures and Tables

**Table 1:** Summary of precision measures that could be utilized in the improvement of feed intake and efficiency, including their relationship with feed intake and efficiency, sensors that collect the precision measures and research involving the precision measure and feed intake and/or efficiency.

Precision Measure	Relationship to Feed Intake and Efficiency	Sensor Types	References
Milk components and metabolites (via MIR ^1^)	Energy requirements Health status	MIR ^1^	Dórea et al., 2018 [34] McParland et al., 2011 [35] McParland et al., 2014 [43] Shetty et al., 2016 [44] Lahart et al., 2019 [45]
Body weight and condition	Maintenance requirements	Image data	Lassen et al., 2018 [39] Shelley et al., 2016 [46] Song et al., 2018 [47] Song et al., 2019 [48] Spoliansky et al., 2016 [49] Zin et al., 2020 [50] Bloch et al., 2019 [51]
Activity	Metabolic rate Health status	Ear tags Rumen boluses Collar mounted Leg mounted	Halachmi et al., 2019 [36] Connor et al., 2013 [52] Hafla et al., 2013 [53] Martin et al., 2021 [54] Olijhoek et al., 2019 [55]
Temperature	Metabolic rate Health status	Ear tags Rumen boluses Thermosensors (vaginal or rectal) Image data	Koltes et al., 2018 [37] Burdick et al., 2012 [56] Fischer et al., 2018 [57]
Rumen health measurements	Health status	Ear tags Collar mounted Rumen boluses	Hamilton et al., 2019 [58]

^1^ Mid-infrared spectroscopy.

## Data Availability

Not applicable.

## References

[1] (2019). Science Breakthroughs to Advance Food and Agricultural Research by 2030.

[2] (2017). National Milk Cost of Production. http://www.ers.usda.gov/data-products/milk-cost-of-production-estimates.aspx.

[3] Food and Agriculture Organization of the United Nations (FAO) (2018). World Food and Agriculture—Statistical Pocketbook.

[4] Knowlton K.F., Radcliffe J.S., Novak C.L., Emmerson D.A. (2004). Animal management to reduce phosphorus losses to the environment. J. Anim. Sci..

[5] von Keyserlingk M.A.G., Martin N.P., Kebreab E., Knowlton K.F., Grant R.J., Stephenson M., Sniffen C.J., Harner J.P., Wright A.D., Smith S.I. (2013). Invited review: Sustainability of the US dairy industry. J. Dairy Sci..

[6] Capper J.L., Cady R.A. (2019). The effects of improved performance in the U.S. dairy cattle industry on environmental impacts between 2007 and 2017. J. Anim. Sci..

[7] Thoma G., Popp J., Nutter D., Shonnard D., Ulrich R., Matlock M., SooKim D., Neiderman Z., Kemper N., East C. (2013). Greenhouse gas emissions from milk production and consumption in the United States: A cradle-to-grave life cycle assessment circa 2008. Int. Dairy J..

[8] (2021). U.S. Dairy Net Zero Initiative. https://www.usdairy.com/getmedia/89d4ec9b-0944-4c1d-90d2-15e85ec75622/game-changer-net-zero-initiative.pdf?ext=.pdf.

[9] Wolfert S., Ge L., Verdouw C., Bogaardt M.-J. (2017). Big Data in Smart Farming—A review. Agric. Syst..

[10] de Souza R.A., Tempelman R.J., Allen M.S., VandeHaar M.J. (2019). Updating predictions of dry matter intake of lactating dairy cows. J. Dairy Sci..

[11] Tempelman R.J., Spurlock D.M., Coffey M., Veerkamp R.F., Armentano L.E., Weigel K.A., de Haas Y., Staples C.R., Connor E.E., Lu Y. (2015). Heterogeneity in genetic and nongenetic variation and energy sink relationships for residual feed intake across research stations and countries. J. Dairy Sci..

[12] Pryce J.E., Gonzalez-Recio O., Nieuwhof G., Wales W.J., Coffey M.P., Hayes B.J., Goddard M.E. (2015). Hot topic: Definition and implementation of a breeding value for feed efficiency in dairy cows. J. Dairy Sci..

[13] Pryce J.E., Wales W.J., De Haas Y., Veerkamp R.F., Hayes B.J. (2013). Genomic selection for feed efficiency in dairy cattle. Animal.

[14] Parish J. (2007). Feedstuff Comparisons—As Fed versus Dry Matter. https://extension.msstate.edu/sites/default/files/topic-files/cattle-business-mississippi-articles/cattle-business-mississippi-articles-landing-page/stocker_feb2007.pdf.

[15] Hardie L. (2016). The Genetic Basis and Improvement of Feed Efficiency in Lactating Holstein Dairy Cattle Recommended Citation. Iowa State University. https://lib.dr.iastate.edu/etd/15926.

[16] Van Der Werf J.H.J. (2004). Is it useful to define residual feed intake as a trait in animal breeding programs?. Aust. J. Exp. Agric..

[17] Veerkamp R.F. (1998). Selection for Economic Efficiency of Dairy Cattle Using Information on Live Weight and Feed Intake: A Review. J. Dairy Sci..

[18] de Haas Y., Calus M.P.L., Veerkamp R.F., Wall E., Coffey M.P., Daetwyler H.D., Hayes B.J., Pryce J.E. (2012). Improved accuracy of genomic prediction for dry matter intake of dairy cattle from combined European and Australian data sets. J. Dairy Sci..

[19] Spurlock D.M., Dekkers J.C.M., Fernando R., Koltes D.A., Wolc A. (2012). Genetic parameters for energy balance, feed efficiency, and related traits in Holstein cattle. J. Dairy Sci..

[20] Vallimont J.E., Dechow C.D., Daubert J.M., Dekleva M.W., Blum J.W., Barlieb C.M., Liu W., Varga G.A., Heinrichs A.J., Baumrucker C.R. (2011). Short communication: Heritability of gross feed efficiency and associations with yield, intake, residual intake, body weight, and body condition score in 11 commercial Pennsylvania tie stalls. J. Dairy Sci..

[21] Armentano L., Weigel K. (2012). Considerations for Improving Feed Efficiency in Dairy Cattle, 1–15. http://dairy.ifas.ufl.edu/rns/2013/1_armentano.pdf.

[22] Connor E.E. (2015). Invited review: Improving feed efficiency in dairy production: Challenges and possibilities. Animal.

[23] Van Arendonk J.A.M., Nieuwhof G.J., Vos H., Korver S. (1991). Genetic aspects of feed intake and efficiency in lactating dairy heifers. Livest. Prod. Sci..

[24] Korver S. (1988). Genetic aspects of feed intake and feed efficiency in dairy cattle: A review. Livest. Prod. Sci..

[25] Kennedy B.W., van der Werf J.H., Meuwissen T.H. (1993). Genetic and Statistical Properties of Residual Feed Intake. J. Anim. Sci..

[26] Koch R.M., Swiger L.A., Chambers D., Gregory K.E. (1963). Efficiency of Feed Use in Beef. J. Anim. Sci..

[27] Hardie L.C., VandeHaar M.J., Tempelman R.J., Weigel K.A., Armentano L.E., Wiggans G.R., Veerkamp R.F., de Haas Y., Coffey M.P., Connor E.E. (2017). The genetic and biological basis of feed efficiency in mid-lactation Holstein dairy cows. J. Dairy Sci..

[28] Ngwerume F., Mao I.L. (1992). Estimation of Residual Energy Intake for Lactating Cows Using an Animal Model. J. Dairy Sci..

[29] VandeHaar M.J., Armentano L.E., Weigel K., Spurlock D.M., Tempelman R.J., Veerkamp R. (2016). Harnessing the genetics of the modern dairy cow to continue improvements in feed efficiency 1. J. Dairy Sci..

[30] Veerkamp R.F., Emmans G.C., Cromie A.R., Simm G. (1995). Variance components for residual feed intake in dairy cows. Livest. Prod. Sci..

[31] Parker Gaddis K.L., VanRaden P.M., Tempelman R.J., Weigel K.A., White H.M., Peñagaricano F., Koltes J.E., Santos J.E.P., Baldwin R., Burchard J. (2021). Implementation of Feed Saved evaluations in the U.S. Interbull Bull..

[32] Wathes C.M., Kristensen H.H., Aerts J.M., Berckmans D. (2008). Is precision livestock farming an engineer’s daydream or nightmare, an animal’s friend or foe, and a farmer’s panacea or pitfall?. Comput. Electron. Agric..

[33] Tullo E., Fontana I., Gottardo D., Sloth K.H., Guarino M. (2016). Technical note: Validation of a commercial system for the continuous and automated monitoring of dairy cow activity. J. Dairy Sci..

[34] Dórea J.R.R., Rosa G.J.M., Weld K.A., Armentano L.E. (2018). Mining data from milk infrared spectroscopy to improve feed intake predictions in lactating dairy cows. J. Dairy Sci..

[35] McParland S., Banos G., Wall E., Coffey M.P., Soyeurt H., Veerkamp R.F., Berry D.P. (2011). The use of mid-infrared spectrometry to predict body energy status of Holstein cows. J. Dairy Sci..

[36] Halachmi I., Guarino M., Bewley J., Pastell M. (2019). Smart Animal Agriculture: Application of Real-Time Sensors to Improve Animal Well-Being and Production. Annu. Rev. Anim. Biosci..

[37] Koltes J.E., Koltes D.A., Mote B.E., Tucker J., Hubbell D.S. (2018). Automated collection of heat stress data in livestock: New technologies and opportunities. Transl. Anim. Sci..

[38] Bezen R., Edan Y., Halachmi I. (2020). Computer vision system for measuring individual cow feed intake using RGB-D camera and deep learning algorithms. Comput. Electron. Agric..

[39] Lassen J., Thomasen J.R., Hansen R.H., Nielsen G.G.B., Olsen E., Stentebjerg P.R.B., Hansen N.W., Borchersen S. Individual measure of feed intake on in-house commercial dairy cattle using 3D camera system. Proceedings of the 11th World Congress of Genetics Applied to Livestock Production.

[40] Fernandes A.F.A., Dórea J.R.R., Rosa G.J.M. (2020). Image Analysis and Computer Vision Applications in Animal Sciences: An Overview. Front. Vet. Sci..

[41] Psota E.T., Schmidt T., Mote B., Pérez L.C. (2020). Long-Term Tracking of Group-Housed Livestock Using Keypoint Detection and MAP Estimation for Individual Animal Identification. Sensors.

[42] Oliveira D.A.B., Pereira L.G.R., Bresolin T., Ferreira R.E.P., Dórea J.R.R. (2021). A review of deep learning algorithms for computer vision systems in livestock. Livest. Sci..

[43] McParland S., Lewis E., Kennedy E., Moore S.G., McCarthy B., O’Donovan M., Butler S.T., Pryce J.E., Berry D.P. (2014). Mid-infrared spectrometry of milk as a predictor of energy intake and efficiency in lactating dairy cows. J. Dairy Sci..

[44] Shetty N., Løvendahl P., Lund M.S., Buitenhuis A.J. (2016). Prediction and validation of residual feed intake and dry matter intake in Danish lactating dairy cows using mid-infrared spectroscopy of milk. J. Dairy Sci..

[45] Lahart B., McParland S., Kennedy E., Boland T.M., Condon T., Williams M., Galvin N., McCarthy B., Buckley F. (2019). Predicting the dry matter intake of grazing dairy cows using infrared reflectance spectroscopy analysis. J. Dairy Sci..

[46] Shelley A.N., Lau D.L., Stone A.E., Bewley J.M. (2016). Short communication: Measuring feed volume and weight by machine vision. J. Dairy Sci..

[47] Song X., Bokkers E.A.M., van der Tol P.P.J., Groot Koerkamp P.W.G., van Mourik S. (2018). Automated body weight prediction of dairy cows using 3-dimensional vision. J. Dairy Sci..

[48] Song X., Bokkers E.A.M., van Mourik S., Groot Koerkamp P.W.G., van der Tol P.P.J. (2019). Automated body condition scoring of dairy cows using 3-dimensional feature extraction from multiple body regions. J. Dairy Sci..

[49] Spoliansky R., Edan Y., Parmet Y., Halachmi I. (2016). Development of automatic body condition scoring using a low-cost 3-dimensional Kinect camera. J. Dairy Sci..

[50] Zin T.T., Seint P.T., Tin P., Horii Y., Kobayashi I. (2020). Body Condition Score Estimation Based on Regression Analysis Using a 3D Camera. Sensors.

[51] Bloch V., Levit H., Halachmi I. (2019). Assessing the potential of photogrammetry to monitor feed intake of dairy cows. J. Dairy Res..

[52] Connor E.E., Hutchison J.L., Norman H.D., Olson K.M., Van Tassell C.P., Leith J.M., Baldwin VI R.L. (2013). Use of residual feed intake in Holsteins during early lactation shows potential to improve feed efficiency through genetic selection. J. Anim. Sci..

[53] Hafla A.N., Carstens G.E., Forbes T.D.A., Tedeschi L.O., Bailey J.C., Walter J.T., Johnson J.R. (2013). Relationships between postweaning residual feed intake in heifers and forage use, body composition, feeding behavior, physical activity, and heart rate of pregnant beef females. J. Anim. Sci..

[54] Martin M.J., Dórea J.R.R., Borchers M.R., Wallace R.L., Bertics S.J., DeNise S.K., Weigel K.A., White H.M. (2021). Comparison of methods to predict feed intake and residual feed intake using behavioral and metabolite data in addition to classical performance variables. J. Dairy Sci..

[55] Olijhoek D.W., Difford G.F., Lund P., Løvendahl P. (2020). Phenotypic modeling of residual feed intake using physical activity and methane production as energy sinks. J. Dairy Sci..

[56] Burdick N.C., Carroll J.A., Dailey J.W., Randel R.D., Falkenberg S.M., Schmidt T.B. (2012). Development of a self-contained, indwelling vaginal temperature probe for use in cattle research. J. Therm. Biol..

[57] Fischer A., Delagarde R., Faverdin P. (2018). Identification of biological traits associated with differences in residual energy intake among lactating Holstein cows. J. Dairy Sci..

[58] Hamilton A.W., Davison C., Tachtatzis C., Andonovic I., Michie C., Ferguson H.J., Somerville L., Jonsson N.N. (2019). Identification of the rumination in cattle using support vector machines with motion-sensitive bolus sensors. Sensors.

[59] Liu E., VandeHaar M.J. (2020). Relationship of residual feed intake and protein efficiency in lactating cows fed high- or low-protein diets. J. Dairy Sci..

[60] Potter T.L., Arndt C., Hristov A.N. (2018). Short communication: Increased somatic cell count is associated with milk loss and reduced feed efficiency in lactating dairy cows. J. Dairy Sci..

[61] Ho P.N., Luke T.D.W., Pryce J.E. (2021). Validation of milk mid-infrared spectroscopy for predicting the metabolic status of lactating dairy cows in Australia. J. Dairy Sci..

[62] Grelet C., Bastin C., Gelé M., Davière J.B., Johan M., Werner A., Reding R., Fernandez Pierna J.A., Colinet F.G., Dardenne P. (2016). Development of Fourier transform mid-infrared calibrations to predict acetone, β- hydroxybutyrate, and citrate contents in bovine milk through a European dairy network. J. Dairy Sci..

[63] Grelet C., Fernández Pierna J.A., Dardenne P., Baeten V., Dehareng F. (2015). Standardization of milk mid-infrared spectra from a European dairy network. J. Dairy Sci..

[64] Grelet C., Fernández Pierna J.A., Dardenne P., Soyeurt H., Vanlierde A., Colinet F., Bastin C., Gengler N., Baeten V., Dehareng F. (2017). Standardization of milk mid-infrared spectrometers for the transfer and use of multiple models. J. Dairy Sci..

[65] Tiplady K.M., Sherlock R.G., Littlejohn M.D., Pryce J.E., Davis S.R., Garrick D.J., Spelman R.J. (2019). Strategies for noise reduction and standardization of milk mid-infrared spectra from dairy cattle. J. Dairy Sci..

[66] Bresolin T., Dórea J.R.R. (2020). Infrared Spectrometry as a High-Throughput Phenotyping Technology to Predict Complex Traits in Livestock Systems. Front. Genet..

[67] Herd R.M., Arthur P.F. (2009). Physiological basis for residual feed intake. J. Anim. Sci..

[68] Lawrence P., Kenny D.A., Earley B., McGee M. (2012). Grazed grass herbage intake and performance of beef heifers with predetermined phenotypic residual feed intake classification. Animal.

[69] Barea R., Dubois S., Gilbert H., Sellier P., van Milgen J., Noblet J. (2010). Energy utilization in pigs selected for high and low residual feed intake. J. Anim. Sci..

[70] Lepron E., Bergeron R., Robert S., Faucitano L., Bernier J.F., Pomar C. (2007). Relationship between residual energy intake and the behaviour of growing pigs from three genetic lines. Livest. Sci..

[71] McPhee C.P., Kerr J.C., Cameron N.D. (2001). Peri-partum posture and behaviour of gilts and the location of their piglets in lines selected for components of efficient lean growth. Appl. Anim. Behav. Sci..

[72] Digiacomo K., Marett L.C., Wales W.J., Hayes B.J., Dunshea F.R., Leury B.J. (2014). Thermoregulatory differences in lactating dairy cattle classed as efficient or inefficient based on residual feed intake. Anim. Prod. Sci..

[73] Britt J.S., Thomas R.C., Speer N.C., Hall M.B. (2003). Efficiency of converting nutrient dry matter to milk in Holstein herds. J. Dairy Sci..

[74] Shuey S.A., Birkelo C.P., Marshall D.M. (1993). The relationship of the maintenance energy requirement to heifer production efficiency. J. Anim. Sci..

[75] Montanholi Y.R., Swanson K.C., Schenkel F.S., McBride B.W., Caldwell T.R., Miller S.P. (2009). On the determination of residual feed intake and associations of infrared thermography with efficiency and ultrasound traits in beef bulls. Livest. Sci..

[76] O’Brien M.D., Rhoads R.P., Sanders S.R., Duff G.C., Baumgard L.H. (2010). Metabolic adaptations to heat stress in growing cattle. Domest. Anim. Endocrinol..

[77] St-Pierre N.R., Cobanov B., Schnitkey G. (2003). Economic losses from heat stress by US livestock industries 1. J. Dairy Sci..

[78] West J.W., Mullinix B.G., Bernard J.K. (2003). Effects of hot, humid weather on milk temperature, dry matter intake, and milk yield of lactating dairy cows. J. Dairy Sci..

[79] Wheelock J.B., Rhoads R.P., VanBaale M.J., Sanders S.R., Baumgard L.H. (2010). Effects of heat stress on energetic metabolism in lactating Holstein cows. J. Dairy Sci..

[80] Derno M., Nürnberg G., Kuhla B. (2019). Characterizing the metabotype and its persistency in lactating Holstein cows: An approach toward metabolic efficiency measures. J. Dairy Sci..

[81] Potts S.B., Boerman J.P., Lock A.L., Allen M.S., VandeHaar M.J. (2017). Relationship between residual feed intake and digestibility for lactating Holstein cows fed high and low starch diets. J. Dairy Sci..

[82] Lam S., Munro J.C., Zhou M., Guan L.L., Schenkel F.S., Steele M.A., Miller S.P., Montanholi Y.R. (2017). Associations of rumen parameters with feed efficiency and sampling routine in beef cattle. Animal.

[83] Geishauser T., Linhart N., Neidl A., Reimann A. (2012). Factors associated with ruminal pH at herd level. J. Dairy Sci..

[84] Krause K.M., Oetzel G.R. (2006). Understanding and preventing subacute ruminal acidosis in dairy herds: A review. Anim. Feed. Sci. Technol..

[85] Plaizier J.C., Krause D.O., Gozho G.N., McBride B.W. (2008). Subacute ruminal acidosis in dairy cows: The physiological causes, incidence and consequences. Vet. J..

[86] Abdela N. (2016). Sub-acute Ruminal Acidosis (SARA) and its Consequence in Dairy Cattle: A Review of Past and Recent Research at Global Prospective. Achiev. Life Sci..

[87] Jami E., White B.A., Mizrahi I. (2014). Potential role of the bovine rumen microbiome in modulating milk composition and feed efficiency. PLoS ONE.

[88] Khiaosa-ard R., Zebeli Q. (2014). Cattle’s variation in rumen ecology and metabolism and its contribution to feed efficiency. Livest. Sci..

[89] Kruger Ben Shabat S., Sasson G., Doron-Faigenboim A., Durman T., Yaacoby S., Berg Miller M.E., White B.A., Shterzer N., Mizrahi I. (2016). Specific microbiome-dependent mechanisms underlie the energy harvest efficiency of ruminants. ISME J..

[90] Mao S., Zhang M., Liu J., Zhu W. (2015). Characterising the bacterial microbiota across the gastrointestinal tracts of dairy cattle: Membership and potential function. Sci. Rep..

[91] Myer P.R., Smith T.P.L., Wells J.E., Kuehn L.A., Freetly H.C. (2015). Rumen microbiome from steers differing in feed efficiency. PLoS ONE.

[92] Sasson G., Ben-Shabat S., Seroussi E., Doron-Faigenboim A., Shterzer N., Yaacoby S., Berg Miller M., White B.A., Halperin E., Mizrahi I. (2017). Heritable Bovine Rumen Bacteria Are Phylogenetically Related and Correlated with the Cow’s Capacity To Harvest Energy from Its Feed. Am. Soc. Microbiol..

[93] Li F., Li C., Chen Y., Liu J., Zhang C., Irving B., Fitzsimmons C., Plastow G., Guan L.L. (2019). Host genetics influence the rumen microbiota and heritable rumen microbial features associate with feed efficiency in cattle. Microbiome.

[94] Petri R.M., Schwaiger T., Penner G.B., Beauchemin K.A., Forster R.J., McKinnon J.J., McAllister T.A. (2013). Changes in the rumen epimural bacterial diversity of beef cattle as affected by diet and induced ruminal acidosis. Appl. Environ. Microbiol..

[95] Wallace R.J., Sasson G., Garnsworthy P.C., Tapio I., Gregson E., Bani P., Huhtanen P., Bayat A.R., Strozzi F., Biscarini F. (2019). A heritable subset of the core rumen microbiome dictates dairy cow productivity and emissions. Sci. Adv..

[96] Ellison M.J., Conant G.C., Lamberson W.R., Cockrum R.R., Austin K.J., Rule D.C., Cammack K.M. (2017). Diet and feed efficiency status affect rumen microbial profiles of sheep. Small Rumin. Res..

[97] Lopes D.R.G., La Reau A.J., Duarte M.S., Detmann E., Bento C.B.P., Mercadante M.E.Z., Bonilha S.F.M., Suen G., Mantovani H.C. (2019). The Bacterial and Funcal Microbiota of Nelore Steers is Dynamic Across the Gastrointestinal Tract and Its Fecal-Associated Microbiota Is Correlated to Feed Efficiency. Front. Microbiol..

[98] Elolimy A., Alharthi A., Zeineldin M., Parys C., Loor J.J. (2020). Residual feed intake divergence during the preweaning period is associated with unique hindgut microbiome and metabolome profiles in neonatal Holstein heifer calves. J. Anim. Sci. Biotechnol..

[99] VandeHaar M.J., St-Pierre N. (2006). Major advances in nutrition: Relevance to the sustainability of the dairy industry. J. Dairy Sci..

[100] de Ondarza M.B., Tricarico J.M. (2017). Review: Advantages and limitations of dairy efficiency measures and the effects of nutrition and feeding management interventions. Prof. Anim. Sci..

[101] Maltz E., Barbosa L.F., Bueno P., Scagion L., Kaniyamattam K., Greco L.F., De Vries A., Santos J.E.P. (2013). Effect of feeding according to energy balance on performance, nutrient excretion, and feeding behavior of early lactation dairy cows. J. Dairy Sci..

[102] NRC (2001). Nutrient Requirements of Dairy Cattle.

[103] Ballou M.A. (2012). Growth and Development Symposium: Inflammation: Role in the etiology and pathophysiology of clinical mastitis in dairy. J. Anim. Sci..

[104] Collier R.J., Renquist B.J., Xiao Y. (2017). A 100-Year Review: Stress physiology including heat stress. J. Dairy Sci..

[105] Kvidera S.K., Horst E.A., Abuajamieh M., Mayorga E.J., Sanz Fernandez M.V., Baumgard L.H. (2016). Technical note: A procedure to estimate glucose requirements of an activated immune system in steers. J. Anim. Sci..

[106] Bauman D.E., McCutcheon S.N., Steinhour W.D., Eppard P.J., Sechen S.J. (1985). Sources of variation and prospects for improvement of productive efficiency in the dairy cow: A review. J. Anim. Sci..

[107] Olson K.M., Cassell B.G., Hanigan M.D., Pearson R.E. (2011). Short communication: Interaction of energy balance, feed efficiency, early lactation health events, and fertility in first-lactation Holstein, Jersey, and reciprocal F1 crossbred cows. J. Dairy Sci..

[108] Bach A., Dinarés M., Devant M., Carré X. (2007). Associations between lameness and production, feeding and milking attendance of Holstein cows milked with an automatic milking system. J. Dairy Res..

[109] González L.A., Tolkamp B.J., Coffey M.P., Ferret A., Kyriazakis I. (2008). Changes in feeding behavior as possible indicators for the automatic monitoring of health disorders in dairy cows. J. Dairy Sci..

[110] Rutten C.J., Velthuis A.G.J., Steeneveld W., Hogeveen H. (2013). Invited review: Sensors to support health management on dairy farms. J. Dairy Sci..

[111] King M.T.M., LeBlanc S.J., Pajor E.A., Wright T.C., DeVries T.J. (2018). Behavior and productivity of cows milked in automated systems before diagnosis of health disorders in early lactation. J. Dairy Sci..

[112] Maltz E. (2020). Individual dairy cow management: Achievements, obstacles and prospects. J. Dairy Res..

[113] Piette D., Norton T., Exadaktylos V., Berckmans D. (2019). Individualised automated lameness detection in dairy cows and the impact of historical window length on algorithm performance. Animal.

[114] Van Hertem T., Viazzi S., Steensels M., Maltz E., Antler A., Alchanatis V., Schlageter-Tello A.A., Lokhorst K., Romanini E.C.B., Bahr C. (2014). Automatic lameness detection based on consecutive 3D-video recordings. Biosyst. Eng..

